# Recommendations for starting a grown up congenital heart disease (GUCH) unit

**DOI:** 10.5935/1678-9741.20150037

**Published:** 2015

**Authors:** Fernando Tadeu Vasconcelos Amaral, Paulo Henrique Manso, André Schmidt, Ricardo Nilson Sgarbieri, Walter Villela de Andrade Vicente, Clovis Carbone Junior, Jane Somerville

**Affiliations:** 1 Hospital das Clinicas da Faculdade de Medicina de Ribeirão Preto da Universidade de São Paulo (HCFMRP-USP), Ribeirão Preto, SP, Brazil.; 2 Hospital das Clinicas da Faculdade de Medicina de Ribeirão Preto da Universidade de São Paulo (HCFMRP-USP), Ribeirão Preto, SP, Brazil and Hospital São Francisco, Ribeirão Preto, SP, Brazil.; 3 Emeritus Professor of Cardiology, Imperial College, London, England.

**Keywords:** Congenital Abnormalities, Heart, Adult

## Abstract

During the last decades, advances in diagnosis and treatment of congenital heart disease have allowed many individuals to reach adulthood. Due mainly to the great diagnostic diversity and to the co-morbidities usually present in this age group, these patients demand assistance in a multidisciplinary facility if an adequate attention is aimed. In this paper we reviewed, based in the international literature and also on the authors’ experience, the structural conditions that should be available for these patients. We highlighted aspects like the facility characteristics, the criteria usually adopted for patient transfer from the paediatric setting, the composition of the medical and para- medical staff taking into account the specific problems, and also the model of outpatient and in-hospital assistance. We also emphasized the importance of patient data storage, the fundamental necessity of institutional support and also the compromise to offer professional training. The crucial relevance of clinical research is also approached, particularly the development of multicenter studies as an appropriate methodology for this heterogeneous patient population.

## Abstract (pt)

Durante as últimas décadas, os avanços verificados no diagnóstico e tratamento das cardiopatias congênitas têm permitido que muitos indivíduos cheguem à idade adulta. Devido principalmente à grande diversidade diagnóstica e também às comorbidades habitualmente presentes nesse grupo etário, esses pacientes necessitam ser atendidos numa unidade multidisciplinar, se o objetivo for proporcionar uma assistência adequada. Neste trabalho revisamos, com base na experiência dos autores e na literatura internacional, as condições estruturais que devem estar disponíveis para esses pacientes. Procuramos ressaltar aspectos como as características da unidade, o critério usualmente adotado para transferência desses pacientes da unidade pediátrica, a composição das equipes médica e paramédica levando em consideração os problemas específicos dos pacientes e também o modelo de assistência ambulatorial e hospitalar. Enfatizamos, ainda, a importância do armazenamento dos dados dos pacientes, a necessidade fundamental de apoio institucional e a importância de oferecer treinamento profissional. A relevância da pesquisa clínica é também abordada, particularmente a importância da confecção de estudos multicêntricos, como uma metodologia apropriada para essa heterogênea população de pacientes.

**Table:** 

**Abbreviations, acronyms & symbols**	
CHD	Congenital heart disease
GUCH	Grow up congenital heart disease

## INTRODUCTION

The remarkable development of paediatric cardiology during the last six decades has been responsible for the fact that up to 90% of children with congenital heart disease (CHD) can now reach adulthood^[1]^. This notable achievement is due to several factors like, first, the increasing paediatric awareness of these defects and diagnostic imaging improvement. The refinement of the invasive treatment techniques, a better understanding of the cardiorespiratory physiology and its application in the postoperative period, particularly in neonates, as well as the awareness of the crucial importance of following these patients after intervention in specialized outpatient clinics also play an important role. It is well recognized that paediatric cardiac surgery is an important landmark in the history of patients with CHD since before its development less than 20% of these children could survive to adult age^[2]^. Now we know that the majority of deaths occur at this age range^[3]^ and that the population of adults is greater than that of children with CHD^[4]^. These patients need special attention in an adequate setting in order that the problems and sequelae intrinsic to the heart defect and also the age related co-morbidities can be well approached.

In most developed countries an organized and efficient structure is available to assist these patients. In Brazil, the development of paediatric cardiology and paediatric cardiac surgery has also been responsible for an increasing number of survivors. In the state of São Paulo, where fifteen fully active centers provide assistance for a population of 44 million people, as well as in others where paediatric cardiology and cardiac surgery have been long instituted, there should be GUCH (grown up congenital heart) services as they have contributed so many survivors to the adult population and made such important contributions to the surgical management of CHD. However, adult CHD units well organized in terms of assistance and research are few. In Ribeirão Preto, cardiac surgery has been offered for complex CHD cases in the last 20 years. The GUCH outpatient clinic started informally about 12 years ago and since 2006 a data base was developed with almost 1000 patients included by now. Difficulties are many in various aspects. As the development of this area among us is mandatory it is thus appropriate to review the needs and recommendations required to establish GUCH specialized services, a population in many areas of the world continues to be neglected. It may not yet be possible to organize a National Service in Brazil so we must be content to concentrate on the region following the **ROCK** principle:

**R**ecognize need

**O**rganise: trying but facing big challenges

**C**entralise facilities and specialists in order not to loose precious resources

**K**nowledge: join specialists and subspecialists - nursing, intensive care, cardiac surgery, complex imaging and anaesthesia altogether.

## INITIAL CONSIDERATIONS

Organization of a GUCH unit must take into account some basic principles applicable to the majority of grown up congenital heart patients:

1) Any paediatric cardiology unit with cardiac surgery should consider the necessity of having a specialized adult cardiac surgery unit for referral, not necessarily on site but within easy travelling. Referral should be activated as soon as the patients reach a certain age between 16 and 19 years depending on maturity and other factors. This transfer of patients should be well planned as many reach adolescence and adulthood without either the family or patient understanding the problems of their defect^[5]^ and need for advice about special situations which appear in adulthood such as physical exercise^[6]^, bacterial endocarditis prophylaxis^[7]^, sexual behavior and pregnancy^[8]^, employment, other systemic diseases, etc. Most have no idea of the future of their lesion and think they are "totally corrected" as doctors and patients have accepted. This requires explanation. Paediatricians and paediatric cardiologists must not lead them into the wilderness of life without explanation or indications of where to go. Loss to follow-up is dangerous for some patients with special conditions. They should be advised, both family and patients.

2) The outpatient demand will increase progressively^[9]^; discharge is rare except for patients with closed ductus or small shunts like atrial and ventricular septal defects, who can be followed out of the referral center^[10]^;

3) The number of patients with complex CHD will increase. The type of lesions seems depend on the group expertise dealing with these cases in the paediatric age throughout the developed world. The lesions in GUCH patients needing medical expertise so are the same;

4) Patients admission for electrophysiological studies^[11]^, cardiac catheterization^[12]^ and surgery^[13]^ to treat sequelae and residual lesions secondary to previous interventions will be more frequent as they growth up.

## LOCAL OF ASSISTANCE

An adequate population estimative must be available to justify costs and presence of experts. Despite different numbers in the literature, it is accepted that a GUCH unit should serve a population of, at least, 4 million people^[3,7]^. Extrapolating Wren's experience in Newcastle, England, based on the number of alive newborns^[14]^, such a number of individuals should generate, in a continuous controlled population, approximately 109 adults with CHD a year. It should be remembered that this population will increase linearly and that demand will naturally increase^[9]^. Another relevant aspect to be considered is the importance to concentrate these cases, mainly the surgical, in a single center, in order to increase the group experience and achieve better results^[15]^. Obviously, in such a huge country as Brazil, where travel difficulty is a reality, we believe that all centers offering invasive treatment for children with CHD should gradually incorporate the assistance to adults. Due to the complexity of the cases, mainly verified in more experienced centers, and also due to patients' age, it is recommended they should be seen in a multidisciplinary unit. Apart from the important occurrence of comorbidities^[16]^, complications and sequelae are frequent, like residual lesions, arrhythmias and pulmonary hypertension^[17]^. Pregnancy can be particularly problematic^[18]^ and a special attention to the mother should be available at the institution. Patients living far away from the regional center could be seen by an interested professional as long as an easy contact with the referral unit is provided^[7]^.

## TRANSFER CRITERIA

Certain lesions do not require automatic transfer to specialized GUCH unit care and this probably makes up 35-40% of the CHD that has been treated in childhood and infancy. Examples are certain ventricular and most atrial septal defects, properly closed ventricular septal defects without murmurs, simple pulmonary valve stenosis and closed ductus. Transfer should be based on patient's age but also taking into consideration his/her psychological and physical maturity^[19]^. Some centers accept patients as early as above twelve years but in the majority of them the age of transfer from paediatric to adult care is sixteen^[20]^. Caring of the adolescent is a particularly important matter which could have serious consequences in adulthood. In some aspects of daily life his behavior and also the relationship with the heart problem is different when compared to adults^[8]^. For this reason we believe that the possible benefit of an outpatient clinic exclusively devoted to this age range should be discussed. Support from adolescent specialists could be helpful preparing theses individuals for adult life.

## STAFF

The composition of the group depends on the number of patients and may vary. Two physicians should be in charge of the first contact with patients, as long as they are interested and have knowledge about CHD and its particularities. There have been some discussion about this matter and some reports even suggest a different kind of professional to deal with these cases^[3]^. In experienced groups there are usually cardiologists and paediatricians^[21]^ who should work in harmony. Specialist imaging must be available to the unit with informed (about GUCH) specialists. At least two surgeons should be available, both with experience in children and adult cases. The anesthetists should also have interest in CHD and have experience in dealing with children and adults. The intensive care physicians in charge of the postoperative care must have an adequate knowledge of CHD in order to understand physiology and its modifications after intervention^[22,23]^. Due to the high incidence of arrhythmias in this population it is advisable that at least one electrophysiologist be a member of the group and have expertise in ablation techniques^[11]^. Cooperation with a pneumology group is desirable due to the significant occurrence of pulmonary hypertension in these patients^[17]^. Despite long-term results are unknown, advanced pharmacologic therapy is available for patients with this complication^[24]^. Also, a good interdisciplinary relationship with a cardiopulmonary transplantation unit with some experience in CHD is necessary. Obstetricians familiar with high risk pregnancies should be available for consultation and assistance. It is recommended that a pathologist, not necessarily working in the same unit, be contacted when necessary. A specialized nurse, as well as a psychologist, a social assistant and secretaries should be part of the group, depending on patient demand. Dental assistance should be provided.

## OUTPATIENT CLINIC

It is well known there is a significant difference between the estimated number of adults with CHD and the actual number seen by the referral centers^[21]^. Most of them are not under routine follow-up^[25,26]^ and were not properly advised after paediatric age^[27]^. This aspect demands special attention of the unit which should search for the patients lost to follow up. The information provided by the referral center regarding availability of the clinic can increase substantially the workload. However, it is ethically correct and should be instituted in order that patients are cared in a specialized center. The model of assistance should be determined considering the demand of patients and the internal logistic of the center. In most centers the clinic usually occurs in specific week days and the consultation should happen in an adequate and comfortable place including the waiting room. Cyanotic and pulmonary hypertensive patients have a poor tolerance to closed and hot places. These patients need to be educated about CHD and should have a private conversation with the physician in order to discuss aspects like sexual behavior, know to be promiscuous in some age ranges^[8]^, genetic risk, pregnancy, exercise^[6]^, contraception, driving and employment^[28]^. Apart from the detailed clinical examination, the electrocardiogram, chest X ray, echocardiogram and hematological tests should be available. More sophisticated tests like cardio exercise test, transeshophagical echocardiography and nuclear ventriculography are necessary for a proper assessment. Magnetic resonance, frequently useful for complex cases^[29]^, it is not routinely available in most of our hospitals but its application is necessary, at least in the referral centers. After patient assessment, a decision has to be made regarding where the follow up is going to occur taking into consideration geographical aspects, individual locomotion and social details. Outreach clinics, a strategy adopted in some places for paediatric cardiology patients and not routinely established in our country, can also be employed for adults with CHD^[30]^. This model of assistance, allowing a specialized consultation, can also be useful to identify those in need of a close follow up^[31]^. The patients requiring intervention should be referred to centers based on the institutional experience and not necessarily to the original patient district. Electronic or telephone consultation between the cardiologists and paediatricians with the referral center should be easy.

## IN HOSPITAL TREATMENT

Due to an increasing number of patients, this is a topic which should be well discussed among each unit aiming to facilitate patient access, which is usually difficult. The number of admissions is directed related to the number of complex cases in follow up. In the more experienced institutions, with an important number of complex CHD cases already treated, admission is common due to reoperations^[28]^ and arrhythmias^[32]^. Also frequent are admissions for interventional catheterization and primary surgery in adults^[33,34]^. Other less frequent causes of admission are endocarditis and high risk pregnancy, which demands specialized obstetrical care^[18]^. These patients should stay in an adult cardiology ward where multidisciplinary assistance is available due to the important incidence of non cardiac clinical and surgical problems^[7,16]^. Centers with high demand should offer a specific ward for these patients^[21]^ which is beneficial for nurse and junior medical experience and training. A crucial aspect is the immediate postoperative care in those with complex CHD particularly when arrhythmias and pulmonary hypertension are present. As well emphasized^[22,23]^ knowledge of pre and post-interventional physiopathological aspects of these defects is essential if proper care is aimed.

## MEDICAL RECORDS

This should be considered of great importance in a GUCH unit. Every patient must be included in a database. The availability of detailed follow up information is mandatory for the patient and for the paediatric group, particularly if any intervention has been performed. Knowledge of the interventions long term results is crucial for improving therapeutic methods and strategies. Due to the great variety of diagnostic and clinical features in GUCH patients the relevance of multicentre studies has been emphasized in the international literature^[34]^ and gathering informations from several centers are needed. The construction of a national database, quite possible in an era of great advances regarding information storage should be an accomplishment to be reached.

## INSTITUTIONAL SUPPORT

It is particularly important that the administrators of tertiary hospitals have some knowledge about the main characteristics of adult patients with CHD as well as the natural history of theses defects and survival possibilities. These data will provide a better resource planning to assist well GUCH patients. The costs of this treatment are usually high mainly when dealing with complex CHD like those with Fontan-type surgery.

## TRAINING

Cardiologists and paediatricians in training must be exposed to the problems faced by adults with CHD, either to learn about specific diagnostic and therapeutic details of the defects or to experience the complexity of an assistance process that usually starts in infancy and reachs adult life, very often with no possibility of cure. In our institution the first consultation of a patient is made by a cardiology resident in his final training period together with the paediatric cardiology resident. As long as training is concerned we believe that the organization of a national net of GUCH services will allow identification of those more suitable for this task. It is recommended that cases requiring intervention be discussed before the procedure in order to achieve therapeutic consensus and training for junior staff.

## RESEARCH

The international literature, mainly in the last decade, has been contemplated by a substantial number of investigations involving adults with CHD. However, the peculiar characteristics of these patients, expressed by a great diagnostic diversity, different residual lesions after intervention as well as the burning of non cardiac diseases, make it difficult to have homogeneous groups of patients with whom basic and clinical research can appropriately be done. Trying to by pass this problem, cooperations have been made between specialized centers in some countries to develop multicenter studies, with statistically reliable patient data and which could answer questions regarding different aspects of adult CHD. A recent report^[35]^ shows a substantial increase in this type of investigation, mainly in the last decade.

It is interesting to mention that, as far as we know, in the last 16 years, only ten papers were published in indexed Brazilian journals regarding CHD in adults^[16,34,36-43]^. However, a change in this perspective is soon expected. The national awareness of the necessity to provide specialized assistance for this group of patients will generate a substantial number of informations. An adequate analysis of these data, either in a single or multicenter investigation will allow the construction of a national database which will make it possible, in a first moment, to know the characteristics of our patients. The planning of specifics investigations using reliable data will then be possible to be elaborated which could, also in a first moment, disclose the results of the paediatric cardiology assistance in the country. The organizations in charge of financing theses researches should be notified about its relevance if the purpose is to provide a proper assistance to this increasing group of patients.

## CONCLUSION

Survival of children with CHD to adult age is a reality in most countries, particularly where good cardiac surgery is available. Paediatric cardiologists and pediatricians should be aware that the great majority of these patients will not be cured and will need follow up for life. They and their parents must be educated since early age and a planned transfer to adult care should be done between 16 and 18 years of age. The follow up of theses adolescents and adults, most of them with sequelaes after intervention should be done in a multidisciplinary institution considering that they will need specific age related attention. Outpatient clinics should be run by interested cardiologists with knowledge of CHD. Percutaneous intervention and heart surgeons with training in complex CHD should be daily available. In hospital treatment should be done in an adult setting with non-invasive and invasive diagnostic resources. Data base storage is essential. Due to the heterogeneous diagnosis verified in this population, research is crucial, including collaborative studies, in order to have good number of patients for data analysis. Junior training is necessary in centers with adequate number of patients and educational facilities. It is necessary that institutions be aware of the peculiar characteristics of these patients, many with complex CHD in need of expensive treatments. We believe that following and adapting theses recommendations to regional care will certainly promote an adequate assistance to the increasing population of GUCH patients. As recently suggested^[44]^ the development of a specific model of assistance is necessary for these patients to minimize risks and offer good treatment.

**Table:** 

**Authors’ roles & responsibilities**	
FTVA	Analysis and/or interpretation of data; final approval of the manuscript; study design; writing of the manuscript or criti-cal review of its content
PHM	Analysis and/or interpretation of data; writing of the manu-script or critical review of its contents
AS	Analysis and/or interpretation of data; final approval of the manuscript; writing of the manuscript or critical review of its content
RNS	Analysis and/or interpretation of data; writing of the manu-script or critical review of its content
WVAV	Analysis and/or interpretation of data; final approval of the manuscript; writing of the manuscript or critical review of its content
CCJ	Analysis and/or interpretation of data; writing of the manu-script or critical review of its content
JS	Analysis and/or interpretation of data; final approval of the manuscript; study design; writing of the manuscript or criti-cal review of its content
